# Burns, Aging, and Appalachia: The Untold Story of Hospital Stays

**DOI:** 10.7759/cureus.77132

**Published:** 2025-01-08

**Authors:** Armein Rahimpour, Ashton R McDonald, Nathan Fox, Alexandra Persily, Willie Kimler, Paul Bown, David Denning, Rahman Barry

**Affiliations:** 1 General Surgery, Marshall University Joan C. Edwards School of Medicine, Huntington, USA; 2 Internal Medicine, Marshall University Joan C. Edwards School of Medicine, Huntington, USA; 3 Plastic and Reconstructive Surgery, Marshall University Joan C. Edwards School of Medicine, Huntington, USA

**Keywords:** burn injury, burn intensive care unit (bicu), burn trauma, rural appalachia, total hospital duration

## Abstract

Background

Burn injuries are among the top 10 causes of trauma and premature death globally. While burns are more prevalent in low-income countries, geriatric burns are disproportionately observed in developed nations, where longer life expectancies result in a larger elderly population. Geriatric burn patients face unique challenges, including slower healing, higher risk of complications, and increased hospital length of stay (LOS), which may be further exacerbated in resource-limited regions such as Appalachia. This study investigates factors influencing hospital LOS in geriatric burn patients aged 65 and older in a resource-constrained region of Appalachia. Variables analyzed include patient demographics, comorbidities, burn-specific characteristics, and the regional healthcare context.

Methodology

This retrospective cohort study analyzed 209 patients admitted to Cabell Huntington Hospital’s Burn Intensive Care Unit between 2017 and 2023. Variables included age, gender, burn severity, total burn surface area (TBSA), inhalation injuries, burn source, comorbidities (chronic obstructive pulmonary disease (COPD), diabetes), smoking history, body mass index, home oxygen use, and discharge status. Statistical analyses included t-tests, analysis of variance, and multivariate regression to identify predictors of LOS.

Results

The mean LOS was 11.7 ± 10.3 days. Factors significantly associated with longer LOS included inhalation injuries (+8.3 days, p = 0.001) and full-thickness burns (+7.9 days, p < 0.001). Patients who died had significantly longer hospital stays (+5.6 days, p = 0.021). Surprisingly, home oxygen use was associated with shorter LOS (-5.1 days, p = 0.011). Furthermore, discharge status was significant. Patients who expired in the hospital had a longer LOS (+5.6 days, p = 0.021). Age, gender, and comorbidities (COPD, diabetes, smoking) did not significantly impact LOS in this cohort. Higher TBSA was associated with longer LOS (p < 0.001). For every 1% increase in TBSA, LOS increased by an average of 0.445 days.

Conclusions

Inhalation injuries, burn severity, and burn source are significant predictors of LOS in geriatric burn patients in Appalachia, while regional resource limitations may uniquely influence care outcomes. Identifying factors affecting LOS can guide tailored interventions to improve recovery and reduce hospital stays in resource-constrained settings. Further research is warranted to explore regional disparities in burn care.

## Introduction

Burn injuries are one of the top 10 causes of trauma and premature death globally [1], resulting from damage and loss of the skin’s protective barrier through high temperatures, electricity, friction, radiation, or chemicals [2]. While burns overall occur more frequently in lower-income countries (estimated 90%), geriatric burns are seen more frequently in developed nations with an incidence of roughly 20% compared to 5% in less developed nations [3]. Developed countries have a longer life expectancy which results in a larger geriatric population. As a result of this, the incidence of geriatric burn injuries is likely to increase [3]. Of the possible etiology of burn injuries in those aged 55 and older, flame injuries were the most common with scald injuries listed as second most common [4]. Geriatric burn patients pose unique challenges regarding their treatment and recovery. Geriatric patients have a less robust healing process due to factors such as thinner atrophic skin, reduced microcirculation, a reduction in epidermal turnover, reduced cardiopulmonary capacity, diminished response to catecholamines, decreased respiratory function, and decreased renal function [5]. For these reasons and the fact that with increasing age comorbidities tend to accumulate, geriatric burn patients exhibit prolonged healing times and have a greater risk for complications to occur which appears to affect hospital length of stay (LOS), with more advanced age being associated with an increase in LOS [1].

The state of West Virginia is located entirely within the Appalachian Mountains which presents unique challenges for patients seeking healthcare. The state is mountainous and largely rural. Rural areas of the United States without widespread access to medical care are frequently referred to as “healthcare deserts,” which are areas where healthcare may not be readily available to residents and tend to be particularly poor in southern West Virginia [6]. Aging rural populations in healthcare deserts may also be prevented from seeking care due to low socioeconomic status, with 16.7% of the population living in poverty as of 2023 [7], as well as challenging mountainous terrain that can limit travel to more resource-rich areas [6]. In addition to limited resources and barriers to travel, a large number of West Virginia’s population suffer from health problems such as high blood pressure, high cholesterol, obesity, and diabetes, with roughly 15% of the population carrying a diagnosis of diabetes, while another 3-4% have diabetes but remain undiagnosed [8]. West Virginia also has a substantial geriatric population, with 21.5% of people over the age of 65 compared to 17.7% nationally [9]. The presence of comorbidities in geriatric burn injuries is a concern because they render elderly individuals more susceptible to complications as well as prolong LOS [5]. Only a few cities in West Virginia, including Morgantown, Charleston, and Huntington, have adequate resources to maintain facilities large enough to address the healthcare needs of the surrounding populations [6]. Of the three cities mentioned above, only Cabell Huntington Hospital in Huntington has a burn intensive care unit (BICU) and can care for burn patients who require an advanced level of care; however, it is still resource-limited with only six beds total. A consequence of this is that burn patients in other parts of the state either have to be transported a long distance to receive care or be transported to an out-of-state facility. The distance from the injury site to a medical facility has an impact on the morbidity of injuries [10], with delays in care potentially prolonging time spent in the hospital away from home.

The purpose of this study is to analyze whether the following characteristics have an effect on LOS in elderly patients located in resource-limited regions such as Appalachia: discharge status, presence of inhalation injuries, degree of the burn, source of the burn, home oxygen use, total burn surface area (TBSA), age, gender, chronic obstructive pulmonary disease (COPD), smoking, and diabetes mellitus (DM). By obtaining a better understanding of which of these characteristics potentially increase LOS, care can be tailored to specific patient needs in an attempt to decrease time spent in the hospital as well as morbidity and mortality.

## Materials and methods

Study design and population

This retrospective cohort study analyzed data from 209 patients aged 65 years and older admitted for burn injuries between January 1, 2017, and January 1, 2023. The study was approved by the Institutional Review Board (approval number: 2063568-1) at Marshall University. Patient records were reviewed retrospectively from the registry at Cabell Huntington Hospital’s Burn Intensive Care Unit. This hospital is an academic teaching hospital, a regional referral center, and an American College of Surgeons-verified Level-2 Trauma Center located in Huntington, West Virginia. The dataset was curated through a formal request to the hospital’s Information Technology (IT) department.

Inclusion and exclusion criteria

The study included all patients aged 65 years and older admitted with burn injuries during the study period. Patients with non-burn conditions such as Stevens-Johnson syndrome, road rash, frostbite, or other unrelated injuries were excluded after manual chart review. A total of 209 patients were included in the final analysis.

Data collection

The following variables were collected for each patient from electronic medical records and the burn registry: (1) demographics: age and gender; (2) clinical variables: a history of COPD, home oxygen use, smoking history (current or former smoker), and DM; (3) burn-specific metrics: source of burn, presence of inhalation injury, body mass index (BMI), TBSA, degree of burn (partial- or full-thickness), and LOS.

The presence of inhalation injury was defined by clinical findings of carbonaceous material or soot in the oropharynx accompanied by difficulty in oxygenation or confirmed through bronchoscopy. TBSA was calculated using the Wallace rule of nines, and the degree of burn was assessed by the attending burn specialist during admission. LOS was defined as the number of days from hospital admission to discharge or death. The primary outcome was LOS in days. Secondary outcomes included the relationship between LOS and demographic, clinical, and burn-specific variables.

Statistical analysis

Descriptive statistics were used to summarize demographic, clinical, and burn-specific variables. Continuous variables were expressed as means with standard deviations, and categorical variables as frequencies and percentages. Differences in LOS across categorical variables were evaluated using independent t-tests or one-way analysis of variance (ANOVA), and associations with continuous variables were assessed using Pearson’s correlation coefficient. Statistical significance was set at p-values <0.05. Univariate linear regression was performed to examine the individual effects of each variable on LOS. Significant variables (p < 0.05) in the univariate analyses were included in a multivariate linear regression model to identify independent predictors of LOS. Outlier detection was performed using the interquartile range (IQR) method. This approach involved calculating the IQR as the difference between the 75th percentile (Q3) and the 25th percentile (Q1). Outliers were defined as any values falling below or above. These values were excluded from the dataset to minimize their potential influence on the regression analysis.

Ethical considerations

This study was conducted in accordance with the principles outlined in the Declaration of Helsinki. The study protocol was approved by the Marshall University Institutional Review Board (approval number: 2063568-1). Due to the retrospective nature of the study, informed consent was waived. All patient data were de-identified to ensure confidentiality.

Software

All statistical analyses were performed using R version 4.2.0 and Python version 3.11. Data visualization was performed using the matplotlib and seaborn libraries.

## Results

A total of 209 patients aged 65 years and older were included in the analysis. The mean age of the cohort was 72.7 ± 5.4 years, and 65.6% (n = 137) of patients were male. The average BMI was 28.8 ± 6.3 kg/m^2^, with 41.6% (n = 87) classified as obese (BMI ≥ 30 kg/m^2^). The mean TBSA burned was 9.6% ± 12.4% for the population without removal of outliers. Inhalation injuries were present in 20.6% (n = 43) of patients. The majority of patients (37.8%, n = 79) had a history of COPD, while 62.2% (n = 130) did not. Additionally, 32.5% (n = 68) used home oxygen, 44.5% (n = 93) were smokers, and 33.3% (n = 69) had DM. The overall mean LOS was 11.7 ± 10.3 days.

Table 1 shows different patient demographics and their relationship to LOS. Gender was not significantly associated with LOS in either univariate or multivariate analyses. Male patients had a slightly higher average LOS of 12.1 ± 10.6 days compared to 11.2 ± 10.0 days in females (t = 1.10, p = 0.271). Discharge status, however, showed a notable impact on LOS. Patients who were discharged alive had an average LOS of 10.9 ± 9.3 days, while those who died during their hospitalization had an average LOS of 16.5 ± 13.7 days (t = 2.56, p = 0.021).

**Table 1:** Patient demographics, LOS, and statistical analysis. The chi-square test was used, and statistical significance was set at p < 0.05. COPD: chronic obstructive pulmonary disease; LOS: length of stay; SD: standard deviation

Characteristic	n (%)	LOS (mean ± SD)	P-value	Test statistic
Gender			0.271	1.1
Female	72 (34.4)	11.2 ± 10.0		
Male	137 (65.6)	12.1 ± 10.6		
COPD			0.465	0.73
Yes	79 (37.8)	12.9 ± 10.8		
No	130 (62.2)	9.7 ± 9.0		
Home oxygen			0.011	-2.57
Yes	68 (32.5)	8.3 ± 7.2		
No	141 (67.5)	13.4 ± 11.3		
Smoking history			0.529	-0.63
Yes	93 (44.5)	11.2 ± 10.2		
No	116 (55.5)	12.1 ± 10.5		
Diabetes			0.464	0.73
Yes	69 (33.3)	12.4 ± 10.7		
No	140 (66.7)	11.4 ± 10.0		
Inhalation injury			0.001	3.44
Yes	43 (20.6)	18.1 ± 13.4		
No	166 (79.4)	9.8 ± 7.8		
Degree of burn			<0.001	5.76
Partial thickness	123 (58.9)	8.3 ± 6.4		
Full thickness	86 (41.1)	16.2 ± 12.5		
Discharge status			0.021	2.56
Alive	30 (14)	10.9 ± 9.3		
Expired	179 (86)	16.5 ± 13.7		

Among the comorbidities, patients with COPD had an average LOS of 12.9 ± 10.8 days compared to 9.7 ± 9.0 days for those without COPD (t = 0.73, p = 0.465). Similarly, patients with diabetes had an average LOS of 12.4 ± 10.7 days compared to 11.4 ± 10.0 days for those without diabetes (t = 0.73, p = 0.464). Smoking history did not show a significant impact on LOS, with smokers and non-smokers exhibiting similar hospital stays (11.2 ± 10.2 days versus 12.1 ± 10.5 days, t = -0.63, p = 0.529). Notably, patients on home oxygen had significantly shorter hospital stays, averaging 8.3 ± 7.2 days compared to 13.4 ± 11.3 days in those not using home oxygen (t = -2.57, p = 0.011).

Patients with inhalation injuries had an average LOS of 18.1 ± 13.4 days compared to 9.8 ± 7.8 days in those without inhalation injuries (t = 3.44, p = 0.001). Patients with higher degrees of burn had significantly longer hospital stays, with full-thickness burns associated with an average LOS of 16.2 ± 12.5 days compared to 8.3 ± 6.4 days for partial-thickness burns (t = 5.76, p < 0.001).

Burn source also significantly influenced LOS. Patients with thermal burns from explosions had an average LOS of 6.8 ± 4.9 days, while those with scald burns from oil or grease had an LOS of 7.1 ± 5.3 days. In contrast, patients with flash burns related to smoking on oxygen had an average LOS of 8.6 ± 6.7 days. These durations were significantly shorter compared to thermal burns from fire (mean LOS 13.9 ± 11.4 days, t = -2.56, p < 0.05).

Table 2 exhibits age not being significantly associated with LOS in either univariate or multivariate analyses. For every one-year increase in age, LOS increased by 0.08 days (t = -0.61, p = 0.543). This effect was small and not statistically significant in the multivariate model.

**Table 2:** LOS and statistical analysis for age, BMI, and TBSA. The t-test was used, and statistical significance was set at p < 0.05. LOS: length of stay; BMI: body mass index; TBSA: total body surface area burned; SD: standard deviation

Characteristic	Mean ± SD	P-value	Test statistic
LOS (days)	11.7 ± 10.3		
Age (years)	72.7 ± 5.4	0.543	−0.61
BMI	28.8 ± 6.3	0.54	0.61
TBSA (%)	5.4 ± 5.02	<0.001	3.596

The multivariate regression analysis demonstrated that several factors independently predicted LOS. Each increase in burn degree was associated with an additional 8.96 days of hospitalization (t = 5.76, p < 0.001). Inhalation injuries independently increased LOS by 6.82 days (t = 3.44, p = 0.001), while home oxygen use reduced LOS by 7.35 days (t = -2.57, p = 0.011). The regression model revealed a statistically significant positive association between TBSA and LOS (t = 3.596, p < 0.001). For every 1% increase in TBSA, LOS increased by an average of 0.445 days. Comorbidities, including COPD and diabetes, did not show statistically significant effects in the multivariate model.

## Discussion

This study analyzed the characteristics and comorbidities of 209 patients admitted to Cabell Huntington Hospital’s BICU from January 1, 2017, to January 1, 2023, with an average LOS of 11.7 ± 10.3 days to analyze which factors significantly contributed to LOS.

Discharge status, referring to whether a patient was discharged from the hospital alive or deceased, had a significant impact on LOS in this study (p = 0.021). Patients who left the hospital alive had an average LOS of 10.9 ± 9.3 days, while patients who were declared deceased while in the hospital had an average LOS of 16.5 ± 13.7 days. On average, a longer LOS among burn patients is associated with increased readmissions and mortality [11] as well as a mortality rate between 3% and 8% [12]. This finding is likely due to the severity of the burns sustained, with patients who had more extensive burns succumbing to their injuries.

Elderly patients are particularly susceptible to developing complications while in the hospital that can contribute to an increased LOS as well as increased mortality. Patients in this age group are more likely to have premorbid conditions such as COPD, diabetes, and cardiovascular disease that can lead to complications such as heart failure, pulmonary edema, and pneumonia [5]. In the event that these patients develop an infection, they are more likely to progress to sepsis and organ failure due to low immunity, reduced organ function reserve, comorbidities such as those mentioned previously, and atypical clinical symptoms of infection leading to missed or incorrect diagnoses [13]. Additionally, elderly patients are more likely to have a dysregulated response to hypermetabolism, which refers to a metabolic state >10% above baseline that attempts to provide near-normal levels of energy to vital organs such as the brain, lungs, heart, and immune organs [14]. This is exacerbated by the fact that elderly patients frequently suffer from micronutrient deficiencies [5] and require additional nutritional support during their hospital stays.

The presence of inhalation injuries in these burn patients had a positive association with time spent in the hospital, contributing to an average LOS of 18.1 ± 13.4 days compared to the LOS of 9.8 ± 7.8 in patients without inhalation injuries (p = 0.021). Inhalation injuries compromise the three main functions of the pulmonary system, i.e., ventilation, oxygenation, and expectoration, and, in doing so, increase the likelihood of suffering poor outcomes [15]. The chemical irritants that enter the lungs during inhalation injuries trigger an inflammatory response within the lungs that leads to bronchoconstriction, increased airway blood flow, and dysregulation of hypoxic vasoconstriction resulting in systemic hypoxemia. Additionally, airway mucosal hyperemia results in tissue edema which prevents air from entering and leaving the lungs. The combination of inflammatory and obstructive effects makes inhalation injuries extremely dangerous and can result in hypoxemia, hypercapnia, acidosis, systemic inflammation, and acute respiratory distress syndrome [15] even if prompt management and treatment are initiated. There are several models for predicting mortality in patients with inhalation injuries, including the abbreviated burn severity index, Belgian Outcome in Burn Injury, Baux score, and revised Baux score (rBaux). The rBaux model has been found to be more accurate than the Baux score in elderly patients with inhalation injuries [16] and is calculated as follows: age (in years) + % surface area burned + (17 × I), where I refers to whether an inhalation injury has been sustained and 0 refers to no inhalation injury [16]. It is important to factor the presence of inhalation injuries into models such as the rBaux score that predicts mortality because of the major impact they have on survival. While TBSA is often the primary factor that influences survival [5], inhalation injuries themselves are associated with mortality rates 20% higher than in patients without inhalation injuries [16] and must also be taken into consideration as a major predictor of mortality.

The degree of burn sustained also had a significant effect on LOS with a notable difference seen between full-thickness and partial-thickness burns (p < 0.001). Full-thickness burns refer to those that involve all layers of the skin and frequently require surgical intervention in the recovery process [2]. The LOS for full-thickness burns in this study was found to be 16.2 ± 12.5 days. Partial-thickness burns can be divided into superficial partial-thickness burns, which only involve the epidermis and superficial layers of the dermis with an average recovery of 10-14 days, and partial-thickness burns that extend deeper into the dermis with a longer recovery period (3-6 weeks) [2]. The average LOS for partial-thickness burns in this study was 8.3 ± 6.4 days.

This study found that the source of the burn was positively associated with LOS in these patients with significance differing by burn type (p < 0.05). Overall, thermal burn injuries are the most common type of burn injury, accounting for roughly 90% of cases [2]. Thermal burn injuries can be further divided into flame injuries, scald injuries, dry heat injuries, and contact injuries, with flame and scald injuries being the first and second most common type of burn in older age groups [2,4]. In this study, the longest LOS was associated with flame burns with an LOS of 13.9 ± 11.4 days, followed by flash burns related to smoking (LOS 8.6 ± 6.7 days). Patients with scald injuries had an LOS of 7.1 ± 5.3 days and the shortest LOS associated with thermal burns from explosions (LOS of 6.8 ± 4.9 days).

Patients who used home oxygen were found to have a significantly shorter hospital stay than patients not on home oxygen (p = 0.011). The average LOS of home oxygen users was 8.3 ± 7.2 days compared to the 13.4 ± 11.3 day LOS of non-home oxygen users. Even though these patients on home oxygen spent a shorter duration in the hospital, on average, those who have suffered a burn injury are twice as likely to have been prescribed supplemental oxygen within the past 90 days compared to those without burn injuries [17]. The oxygen supplied through a face mask or nasal cannula increases the risk of smokers on oxygen therapy sustaining a burn injury, with smoking being the most common cause of burn injury in long-term home oxygen users [18]. Despite the high risk of burn injury in smokers using home oxygen, the decreased LOS seen in this study may be due to home oxygen correcting hypoxemia and improving pulmonary function, as seen in a previous study by Tanash et al [18].

The last significant finding discussed in this study is TBSA, which revealed a statistically significant positive association between TBSA and LOS (p < 0.001). For every 1% increase in TBSA, LOS increased by an average of 0.445 days. TBSA can also influence the risk of developing complications such as severe hypovolemia (burn shock) when TBSA is >30% [2]. TBSA has previously been found to be a significant factor in hospital LOS [19].

The age range of patients included in this study was 72.7 ± 5.4 years with every one additional year in age increasing the LOS by 0.08 days (p = 0.543). This slight increase in LOS is not a significant finding which is surprising because increased age is associated with prolonged healing times and an increased risk of developing comorbidities [5]. Advanced age has previously been found to result in an increased LOS [1]. Future studies might focus on whether this is a finding unique to Appalachian burn patients as a whole or if the findings are limited to the specific patients included in this study.

Several patient comorbidities that were analyzed and did not contribute to an increase in LOS were COPD (p = 0.465) with an LOS of 12.9 ± 10.8 days, smoking history (p = 0.529) with an LOS of 11.2 ± 10.2 days, and DM (p = 0.464) with an LOS of 12.4 ± 10.7 days. Even though these comorbidities did not impact LOS in this study, they still disproportionately affect Appalachian populations. Appalachian populations are at a higher risk for developing COPD, facing hospitalization, and suffering mortality given that nearly 40% of Appalachian counties have COPD mortality rates in the worst-performing national quintile [20]. Central Appalachia, in particular, has the highest percentage of smokers in the nation with 25.2% reporting tobacco use compared to 16% nationally [21]. DM remains a major problem in West Virginia, with one of the highest diabetes incidences in the nation, and an estimate that 17% of the state’s population will carry a diabetes diagnosis by 2025 [22].

Of the 209 patients included in this study, 65.6% were males and 34.4% were females. Gender did not affect LOS in a meaningful way in this study, with males having a slightly higher LOS of 12.1 ± 10.6 compared to the LOS of 11.2 ± 10.0 in females (p = 0.271). Despite gender not being a predictor of LOS in these patients, burn injuries in developed nations are overall more common in men [3]. This holds true up until the oldest age groups when the trend reverses. A study by Pham et al. [4] demonstrated that in the 65-74-year age bracket there was a female predominance in burn injuries (1.25:1) compared to a male-to-female ratio of 1.4:1 in younger age groups. This can be explained by a larger number of women in this age group even with an unchanged total number of male burn patients [4,5].

Although this novel study is unique and fills the gap in understanding Appalachian elderly burn victims, it is limited to being retrospective and a single-institution study. The retrospective design of this study allows for biases and limits generalizability. This population is really unique. Future studies should be performed in a multicenter prospective design to diversify the population and allow for improved generalization.

## Conclusions

This study highlights the significant predictors of hospital LOS in geriatric burn patients aged 65 and older within a resource-limited Appalachian region. Factors such as inhalation injuries, full-thickness burns, flame burn injuries, TBSA, and discharge alive from the hospital were associated with prolonged LOS, while home oxygen use was unexpectedly linked to shorter hospital stays. The findings underscore the unique challenges faced by elderly burn patients in rural and underserved areas, where limited healthcare resources, geographic barriers, and socioeconomic disparities may exacerbate care outcomes. Understanding these predictors allows for more tailored interventions aimed at reducing LOS, improving recovery, and addressing systemic healthcare disparities in Appalachia. Further research is needed to evaluate these findings in larger cohorts and explore strategies for optimizing burn care in resource-constrained settings.

## References

[1] Onah CN, Allmendinger R, Handl J, Dunn KW (2021). Surviving burn injury: drivers of length of hospital stay. Int J Environ Res Public Health.

[2] Żwierełło W, Piorun K, Skórka-Majewicz M, Maruszewska A, Antoniewski J, Gutowska I (2023). Burns: classification, pathophysiology, and treatment: a review. Int J Mol Sci.

[3] Dissanaike S, Rahimi M (2009). Epidemiology of burn injuries: highlighting cultural and socio-demographic aspects. Int Rev Psychiatry.

[4] Pham TN, Kramer CB, Wang J, Rivara FP, Heimbach DM, Gibran NS, Klein MB (2009). Epidemiology and outcomes of older adults with burn injury: an analysis of the National Burn Repository. J Burn Care Res.

[5] Keck M, Lumenta DB, Andel H, Kamolz LP, Frey M (2009). Burn treatment in the elderly. Burns.

[6] Hong I, Wilson B, Gross T, Conley J, Powers T (2023). Challenging terrains: socio-spatial analysis of Primary Health Care Access Disparities in West Virginia. Appl Spat Anal Policy.

[7] (2024). United States Census Bureau. Income and poverty. https://data.census.gov/profile/West_Virginia?g=040XX00US54#income-and-poverty.

[8] Baus A, Shawley-Brzoska S, Wright J (2021). Informatics-supported diabetes prevention programming in West Virginia. Perspect Health Inf Manag.

[9] (2024). United States Census Bureau. Populations and people. https://data.census.gov/profile/West_Virginia?g=040XX00US54#populations-and-people.

[10] Viradia R, Annie FH, Kali M, Pollock F, Hayes JD (2021). Hand injuries of coal miners in southern West Virginia: a pilot study on health-care resources in southern west virginia. J Emerg Trauma Shock.

[11] Han TS, Murray P, Robin J, Wilkinson P, Fluck D, Fry CH (2022). Evaluation of the association of length of stay in hospital and outcomes. Int J Qual Health Care.

[12] Ghaed Chukamei Z, Mobayen M, Bagheri Toolaroud P, Ghalandari M, Delavari S (2021). The length of stay and cost of burn patients and the affecting factors. Int J Burns Trauma.

[13] Zhang L, Huang T, Xu F, Li S, Zheng S, Lyu J, Yin H (2022). Prediction of prognosis in elderly patients with sepsis based on machine learning (random survival forest). BMC Emerg Med.

[14] Zhang P, Zou B, Liou YC, Huang C (2021). The pathogenesis and diagnosis of sepsis post burn injury. Burns Trauma.

[15] Foncerrada G, Culnan DM, Capek KD (2018). Inhalation injury in the burned patient. Ann Plast Surg.

[16] Lam NN, Minh NT (2022). Risk factors for death and prognosis value of revised Baux score for burn patients with inhalation injury. Ann Burns Fire Disasters.

[17] Sharma G, Meena R, Goodwin JS, Zhang W, Kuo YF, Duarte AG (2015). Burn injury associated with home oxygen use in patients with chronic obstructive pulmonary disease. Mayo Clin Proc.

[18] Tanash HA, Ringbaek T, Huss F, Ekström M (2017). Burn injury during long-term oxygen therapy in Denmark and Sweden: the potential role of smoking. Int J Chron Obstruct Pulmon Dis.

[19] Moore RA, Popowicz P, Burns B (2025). Rule of Nines. https://www.ncbi.nlm.nih.gov/pubmed/30020659.

[20] Stellefson M, Wang MQ, Balanay JA, Wu R, Paige SR (2020). Latent health risk classes associated with poor physical and mental outcomes in workers with COPD from central Appalachian U.S. states. Int J Environ Res Public Health.

[21] Cardarelli K, Westneat S, Dunfee M, May B, Schoenberg N, Browning S (2021). Persistent disparities in smoking among rural Appalachians: evidence from the Mountain Air Project. BMC Public Health.

[22] Misra R, Fitch C, Roberts D, Wright D (2016). Community-based diabetes screening and risk assessment in rural West Virginia. J Diabetes Res.

